# The Shape Memory Properties and Actuation Performances of 4D Printing Poly (Ether-Ether-Ketone)

**DOI:** 10.3390/polym14183800

**Published:** 2022-09-11

**Authors:** Yuting Zhou, Luquan Ren, Jianfeng Zang, Zhihui Zhang

**Affiliations:** 1 The Key Laboratory of Bionic Engineering (Ministry of Education), The College of Biological and Agricultural Engineering, Jilin University, 5988 Renmin Street, Changchun 130025, China; 2 School of Optical and Electronic Information and Wuhan National Laboratory for Optoelectronics, Huazhong University of Science and Technology, Wuhan 430074, China; 3 The State Key Laboratory of Digital Manufacturing Equipment and Technology, Huazhong University of Science and Technology, Wuhan 430074, China

**Keywords:** 4D printing, shape memory polymers (SMPs), polyether-ether-ketone (PEEK), actuation performances, deployable structure

## Abstract

Shape-memory polymers (SMPs) have gradually emerged in the mechanism and biomedical fields and facilitate the upgrading of industrial mechanisms and the breakthrough of technical bottlenecks. However, most of the SMPs are infeasible in harsh environments, such as aerospace, due to the low glass transition temperature. There are still some works that remain in creating truly portable or non-contacting actuators that can match the performances and functions of traditional metal structures. Polyether-ether-ketone (PEEK) with a high glass transition temperature of 143 °C is endowed with outstanding high-temperature resistance and radiation-resistant properties and shape memory behavior. Thus, we explore the shape-memory properties and actuation performances of high-temperature PEEK in bending behaviors. The shape-recovery ratio, actuation speed and force under different programming conditions and structure parameters are summarized to complete the actuation capacities. Meanwhile, a metallic ball transported by shape-memory PEEK and deployed drag sail with thermo-responsive composite joints were shown to verify the potential in aerospace.

## 1. Introduction

Thermally-induced shape-memory polymers (SMPs) have the capability to be fixed into a temporary shape when exposed to external thermal stimuli and then recover to their original shape at a high temperature [1,2,3]. Due to this active deformability, known as the shape memory effect [4], SMPs gradually play a role in mechanism [5,6,7,8] and biomedical fields [9,10,11,12], and facilitate industrial upgrading and breakthroughs of technical bottlenecks. Despite impressive progress for SMPs emerging recently, such as occlusion devices for heart diseases [13], robotic fingers for grasping action [14], and digital logic circuits for programmable mechanical computing [15], the low glass transition temperature (T_g_) limits the functionality in harsh environment. There are still some works that remain in creating truly portable or non-contacting SMPs actuators that can match the performances and functions of traditional mechanical structures.

Polyether-ether-ketone (PEEK) is a special engineering plastic and also has a certain shape memory effect. The melting temperature of PEEK is 343 °C, and the T_g_ is 143 °C [16], which is higher than the typical temperature of the Low Earth Orbit, going from −100 °C to 120 °C [17]. Namely, the shape memory effect of PEEK can work in the Low Earth Orbit. Moreover, the unique high-temperature resistance and radiation-resistant properties [18,19] endow PEEK with a more extensive prospect than other SMPs as an aerospace material. Existing research for PEEK mainly aims at enhancing its mechanical properties [20,21,22,23], such as chemical modification [24] and fiber-reinforced integration [25,26]. Though the shape memory effect of PEEK was characterized in a previous study under small strain with tensile mode [27], bending rather than tension is the main actuation method in the applications of SMPs [28,29,30]. Therefore, the shape memory effect and actuated capacities in bending deformation with large strain are considered noteworthy properties for PEEK.

In most studies of SMPs, researchers concentrate on the shape fixed ratio or the recovery ratio of the SMPs and try to promote them for expanding the applications [31,32]. Fewer researchers study the actuation characteristics (speed and force), even though the shape memory behavior enables SMPs a great potential for actuation. On the one hand, the SMPs are considered to have a slower recovery speed than that of shape memory alloys (SMAs). However, the controllable strain of SMAs is only 8% [33], which is much smaller than that of SMPs (up to 800%) [34], and the deformation capacity of SMAs can only be improved by restricted structural design [35]. The SMA actuator is wire [36] with a limited diameter and size such as a spring-like structure [37,38]. When the strain of actuators is satisfied, the structure is specified, namely, integrated structure-function devices are hard. Moreover, conventional SMAs are limited to maximal A_f_-temperatures of 120 °C, M_s_ being below 100 °C [39], and are not suitable for aerospace as mentioned before. Therefore, SMPs play an important role in smart actuators all the time. On the other hand, the stimuli required for shape memory deformation are difficult to provide. Most of SMPs are thermo-responsive and need a high-temperature environment [40,41], which limits the test methods. Four-dimensional printing combined 3D printing with SMPs, bringing a new dimension, time, for 3D printed constructs [42]. Four-dimensional printing induced diversely designed structures for SMPs to gain further application by being responsive to stimuli [43]. We combine materials and stimuli by 4D composite printing in our preview study [44] and replace the indirect heating (hot air or oil) with a contact source embedded in SMPs.

In this work, the shape memory properties and actuation performances of high-temperature PEEK were explored in bending behavior. Firstly, the 4D printing PEEK composites embedded with excitation source were printed. Secondly, the shape memory behavior and actuation capacities under different programming conditions (recovery temperatures, cooling speeds, and idling time) and structure parameters (bending arc length and thickness) were summarized. Finally, we demonstrated the lift and transport of a metallic ball by shape memory PEEK. At the same time, an imitative deployable drag sail and catcher were developed to illustrate the extensive potential. The studies are expected to provide a basic characteristic for shape memory PEEK and pave the way toward novel applications ranging from the shape memory behavior of PEEK to deployable structures in aerospace.

## 2. Materials and Method

### 2.1. Materials and 4D Printing Method

**Figure 1:**
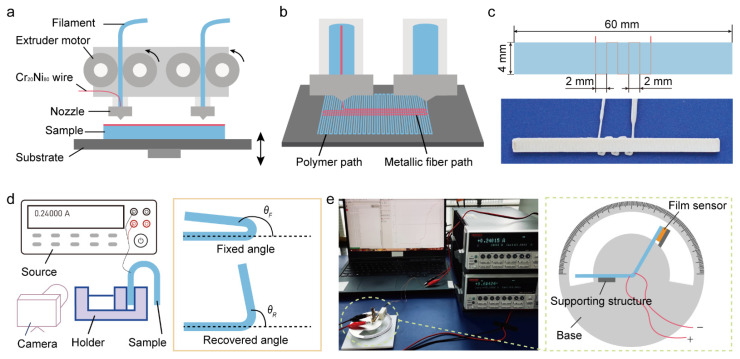
Four-dimensional printing and test method of shape memory PEEK. (**a**) The equipment and construction of 4D printing. (**b**) The process of 4D printing PEEK composites. (**c**) The printed composite sample and sizes. (**d**) The measurement method and definition of angle. *θ_F_* was the fixed angle and *θ_R_* was the recovered angle between the horizontal line and the free side of the sample. (**e**) The test method and devices of the recovery force.

We used a modified double-extruders 3D printing device to prepare the PEEK samples shown in Figure 1a. Firstly, one heating extruder (380 °C) equipped with PEEK filament (Joinature Polymer Co., Ltd., Changchun, China) successively printed the layers of PEEK base on the hot substrate (110 °C). Then, the other extruder embedded with continuous wire printed on the surface of the PEEK base, configuring a continuous conductive path, illustrated in Figure 1b. We chose the widespread Cr_20_Ni_80_ wire (Juanhui Technology Co., Ltd., Hangzhou, China) with a diameter of 0.05 mm as the continuous wire because of its steady electrothermal effect, which can offer a thermal stimulus for shape memory effect. The properties of the Cr_20_Ni_80_ wire were described in Table S1. The continuous conductive path could be customized by the distribution density or position for the required temperature gradient or deformation areas. Finally, the continuous conductive path was covered with layers of the PEEK base the same as before. These composite samples combined the material and stimulus, enabling the shape memory PEEK to transform and recover anywhere without a thermal container. More details and the principle of 4D printing PEEK composites were explained in our previous study [44]. The printed sandwich structure composite shown in Figure 1c consisted of PEEK bases with five layers at the bottom and top, respectively, and a one-layer wire path in the middle. The pattern of the Cr_20_Ni_80_ wire embedded in the PEEK base was printed with a printing speed of 150 mm/min and a printing height of 0.1 mm. The continuous wire was distributed into the “S” path, and adjacent wires were spaced 2 mm apart. The effective heating area was regarded as 10 mm × 4 mm.

### 2.2. Design and Experiments

Shape-fixed ratio and shape-recovery ratio were two significant indexes that represented the working potential for thermo-responsive shape memory PEEK. Therefore, we studied the macroscopical shape memory properties of PEEK in bending behavior. The shape recovery of PEEK was recorded by a camera, and a program that could identify the variational angle values frame by frame was written in Python 3.10. The test devices and method were demonstrated in Figure 1d. The flat sample was locally heated up by the power wire connected with a source (APS3005S-3D, GRATTEN, ATTEN Technology Co., Ltd., Shenzhen, China), while the flexible sample was shaped by applied strain. After cooling down, the sample kept a temporary shape with a fixed angle *θ_F_*, which was the angle between the horizontal line and the free side of the sample. Thus, we could calculate the shape fixed ratio *R_f_* according to Equation (1). Then the temporary shape was heated up again and recovered to a permanent shape with a recovery angle *θ_R_*. We could get the shape recovery ratio *R_f_* from Equation (2). These two significant indexes characterized the shape memory effect of the PEEK. The larger fixed ratio meant the more accurate control for temporary shape, and the larger recovery ratio meant the more outstanding capability to recover the original shape.

(1) $$R_{f}=\frac{\theta_{F}}{180}\times 100\%$$

(2) $$R_{r}=\frac{\theta_{F}-\theta_{R}}{\theta_{F}}\times 100\%$$

The key parameters of actuation performances were actuation speed and actuation force. The actuation speed represented the deformation efficiency, while the actuation force, namely the recovery force, indicated the capability of work output. The polynomial fitting with eighth-order was applied to the angle curve in software Origin, and the recovered angle speed was the derivative of the fitting curve of the angle. The recovery force was measured by a customized processing holder with an angle scale in Figure 1e and Video S1. The composite PEEK sample was triggered by a power wire, while a pressure sensor (Yubo Intelligent Technology Co., Ltd., Hangzhou, China) fixed on the supporting structure was connected with a source measure unit (Keithley 2400, Tektronix Inc., Beaverton, OR, USA) to successively detect the pressure. The following experiments were implemented with 4D printed PEEK composites by the above method, and every test was repeated 3 times.

## 3. Result and Discussion

### 3.1. The Cycle Shape Memory Properties and Actuation Performances of PEEK

**Figure 2:**
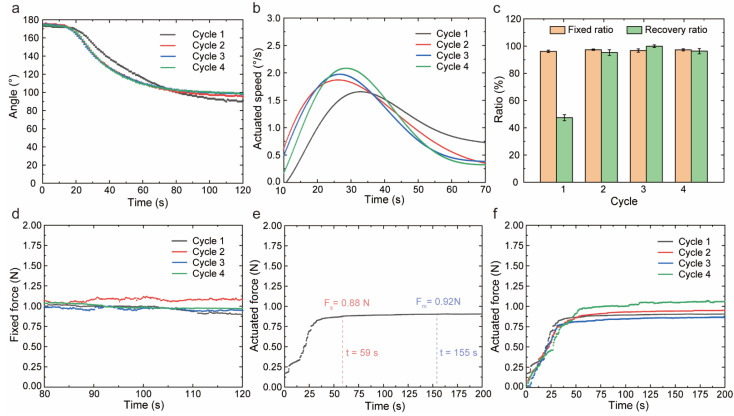
The cycle shape memory properties and actuation performances of PEEK. (**a**) The variation in angle in cycle shape recovery process. (**b**) The angular speed in real-time for four cycles. (**c**) The shape fixed ratio and shape recovery ratio in cycle shape memory behavior. (**d**) The fixed force in cycle shape fixed process. (**e**) The recovery force in real-time. *F_s_* was the steady force and *F_m_* was the maximum force. (**f**) The recovery force in the cycle shape recovery process.

We studied the shape memory effect of PEEK under full-bending deformation (180°) at the actuated current of 0.24 A. In several recovery cycles, the successive values of angle and speed with time were described in Figure 2a,b. The overall performances of the recovery process were described as follows: (i) after 15 s of power-on, the temperature reached 170 °C and obvious recovery occurred; (ii) the recovered angle increased rapidly within 10 s, while the maximum angular speed showed at 25–30 s; (iii) then the angular speed slowed down gradually, and the change in the angle was insignificant at 70th second; (iv) after 70 s, the recovery was regarded as a stable relaxation at high temperature. In multiple cycles of shape memory bending, the first cycle manifested the distinct response. The recovered angle and angular speed were smaller than that of the other cycles at the same time. The maximum angular speed of the first cycle was 1.655 °/s, which was 0.84 times that of other cycles. With the slower speed equipped, the recovery behaviors of the first cycle lasted for a longer time. The recovered angle of 82° was larger than other cycles, and the shape recovery ratio was 47%. Through the curve of angular speed, the first cycle showed a wider peak, and the recovery time (90 s) was 1.8 times that of other cycles. According to the records of angle, we calculated the fixed ratio and recovery ratio for shape memory cycles in Figure 2c. The fixed ratio maintained above 96%, while the recovery ratio stayed above 95% except for the first cycle. We considered that the different behaviors of the first cycle were caused by these two reasons. For the angle, the high temperature in the first recovery meant using an anneal process to produce tiny crystals in the heating region and increase the crystallinity of PEEK. Furthermore, tiny crystals served as the physical cross-link points, which could restrict the slip of chains during the mechanical deformations in the following cycles. In this condition, more input energy was applied to elastic deformation rather than plastic deformation induced by molecular chain slippage. Therefore, more recoverable strain energy was stored and triggered off a higher shape recovery ratio in the following recovery process. For the angular speed, tremendous bending at a high temperature gave rise to the change in microstructure. The layers pressed against each other under tensile stress, hence, the small holes induced by the 4D printing process were eliminated, and the layers became tighter resulting in a higher heat transfer rate in the preheating steps.

The actuation capacities of PEEK in shape memory cycles were demonstrated in Figure 2d–f. When the samples were bent at a high temperature, the applied force, defined as fixed force, was recorded in Figure 1d. During the fixed process, the fixed force stabilized at 1.02 N after a while. Moreover, the recovered force was tested as a key indicator, which determined the actuation capacity. Figure 2e showed a whole force curve of the recovery process in the first cycle. The force increased fast until the 35th second, which corresponded to the maximum angular speed. When the speed decreased, the force showed a slow change. After the 59th second, the force kept stable at 0.88 N, which was 95% of the maximum force value (0.92 N). The shape memory actuation of PEEK lasted about 155 s, and the main actuation occurred in the first 23% of the time. The recovered force was consistent across multiple cycles shown in Figure 2f.

To verify the stable shape memory effect of PEEK, we conducted consecutive shape memory cycles 50 times. For the 50 cycles, the angular recovery and corresponding speed were accordantly shown in Figure S1a,b. The average recovered angle was 61.6°, and the average maximum speed was about 1.69 °/s. According to Figure S1c,d, the average fixed force was 1.13 N with a standard deviation of 0.03 N, while the average recovery force was 1.06 N with a standard deviation of 0.04 N. It followed that the working capacities and deformation accuracy of PEEK actuators were reliable for dozens of actions. Moreover, the surface topography of PEEK after 50 cycles was observed by a microscope (Alpha300 R, WITec, Ulm, Germany) in Figure S2a,b. As can be seen from the figure, the surface that had been repeatedly heated and bent was the same as the original surface. Moreover, there was no separation between layers in the thickness direction in Figure S2c. These verified the reliability of PEEK in multiple shape memory deformation.

### 3.2. The Effect of Temperature on Shape Memory Properties and Actuation Performances

**Figure 3:**
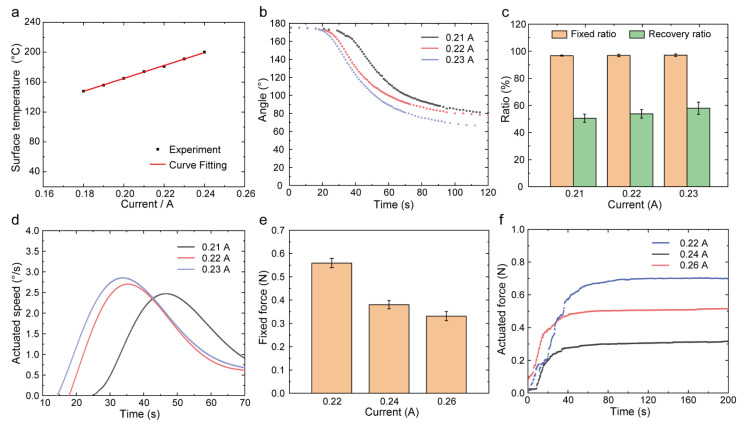
The effect of currents on shape memory properties and actuation performances. (**a**) The relationship between the surface temperature of samples and currents of wire. (**b**) The variation in angle for different currents in the recovery process. (**c**) The angular speed for different currents. (**d**) The shape memory properties for different currents. (**e**) The fixed force for different currents. (**f**) The recovery force for different currents.

Shape memory recovery was a relaxation process [45]. When the shape was fixed with applied force or strain under high temperature, the elastic deformation was held at the rubber state. The input energy was frozen as strain energy with the temperature cooling down, while the temporary shape was fixed. The fixed shape was not an eternal state, and the stored energy would release with the time elapsed because of the stress relaxation. Therefore, the shape would gradually recover to the original shape even without stimulus (high temperature). The relaxation was a time-dependent process. Increasing the temperature was equivalent to increasing the time, so the recovery was supposed to be a temperature-dependent deformation. Based on this, we investigated the shape memory effect of PEEK under full-bending deformation (180°) at different actuated currents. The actuated currents and surface temperature of the PEEK samples had a rigorous linear relationship shown in Figure 3a, while the thermal imaging maps recorded by FLIR T420 under different currents were shown in Figure S3. According to the curves of angle in Figure 3b, the currents had an apparent effect on the recovered angle. The higher current resulted in earlier recovery. When the current increased by 0.02A, corresponding to a 20 °C increase in temperature, the preheating time was reduced by 10 s. The temperature of the samples reached above the glass transition temperature faster due to a higher heating rate, appearing as the curves shifted to the left. At the end of recovery, the higher current led to a larger recovered angle and better shape recovery ratio, shown in Figure 3c. This was consistent with the previous theory. The relationship between heating temperature (*T*) and recovery ratio was analyzed by linear regression analysis with R^2^ = 0.999, and shown as Equation (3). Additionally, the maximum angular speed increased with current, shown in Figure 3d. When the current increased from 0.21 A to 0.23 A, the recovery ratio increased by 14% (from 50.5% to 57.9%), and the maximum speed increased by 10% (from 2.38 °/s to 2.63 °/s). The relationship between heating temperature and recovery speed (*V*) was analyzed by linear regression analysis with R^2^ = 0.997, and shown as Equation (4).

(3) $$R_{r}=0.4T-24.7$$

(4) V=0.014T−0.119

When a higher current was applied, the sample became softer due to the high temperature, so the fixed force appeared weak; shown in Figure 3e. However, the recovery force under different currents showed diverse laws in Figure 3f. The current of 0.22 A resulted in a maximum recovery force, but the current of 0.24 A contributed to a minimum recovery force. The actuated force decreased by 0.2 N for every 20 °C increase in temperature. This was interpreted as the coupling of two factors - hardness and acceleration. The softer the material would result in a lower output force. But we could see from Figure 3d that the greater the current, the greater the acceleration. Therefore, a larger current generated a larger recovery force. We conducted the one-way ANOVA for repeated measures of shape memory properties and actuation performances in Origin. The F-value and *p*-value were shown in Table S2. Recovery ratio and actuated speed insignificantly increased when the current increased (*p* > 0.05) due to the limited temperature difference and the nonnegligible deviation of fixed ratio. The fixed force and actuated force were significantly lower when the current increased (*p* < 0.05).

### 3.3. The Effect of Cooling Speed on Shape Memory Properties and Actuation Performances

**Figure 4:**
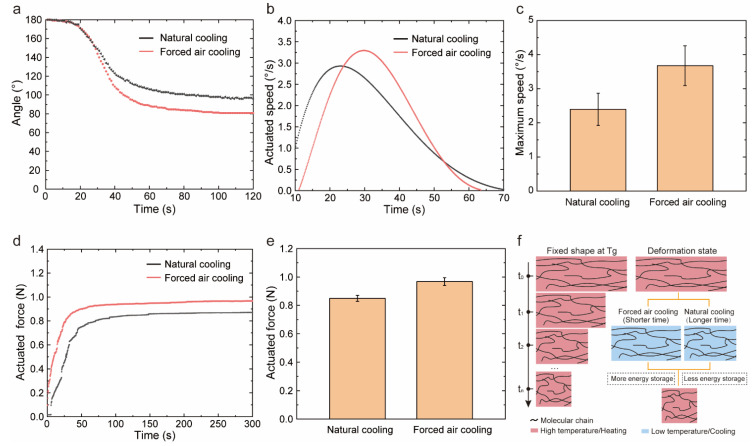
The effect of cooling speeds on shape memory properties and actuation performances. (**a**) The variation in angle for different cooling speeds. (**b**) The angular speed for different cooling speeds. (**c**) The maximum speed for different cooling speeds in the recovery process. (**d**) The recovery force for different cooling speeds. (**e**) The maximum recovery force for different cooling speeds. (**f**) The schematic diagram of effect for different cooling speeds.

The shape memory behavior included the fixation of temporary shape and the recovery of permanent shape and was determined by the fixed and recovery process. In the previous section, the temperature in the recovery process had been changed to adjust the shape memory properties and actuation performances of PEEK. Here, we continued to study the effect of cooling speed in the fixed process. Natural cooling and forced air cooling were applied to the sample, respectively, while the samples were constrained at 180°. From Figure 4a, the fixed ratio was the same for both cooling conditions, but the recovery ratio appeared different. Forced air cooling led to a higher recovery ratio (58%) than that of natural cooling (49%) because of the faster speed in Figure 4b,c. Under forced air cooling, the recovery speed (3.3 °/s) was increased by 1.5 times. Moreover, the forced air cooling enhanced the recovery force by 14%, and the enhancement effect was maintained throughout the whole process shown in Figure 4d,e. The improvement of shape memory and actuation performance by forced air cooling was attributed to the freeze of the strain energy illustrated in Figure 4f. When the samples were bent at T_g_, the long chains in distorted areas were stretched. The elongated chains would spontaneously restore to the tangled state at the high temperature due to the entropic elasticity. However, because of the viscidity, this elastic recovery was a time-dependent process with time series t. Assuming that the initial state of the relaxation process corresponded to t_0_ and the final state corresponded to t_n_, while there were intermediate states corresponding to t_1_ and t_2_. When the sample was cooled down by the forced air, namely short cooling time, the sample recovered to t_1_ due to the elasticity. However, when the sample was cooled down naturally, namely long cooling time, there was sufficient time for the sample to recover to t_2_. Thus, the samples with different cooling strategies would freeze different strain energy. A small cooling speed would offer SMPs more time to evolve towards the equilibrium state, which shifts the onset of the recovery process to a higher temperature [46]. In conclusion, a small cooling speed induced a slower shape recovery behavior and weaker actuated capacity during heating at the given condition. Furthermore, we conducted the one-way ANOVA for shape memory properties and actuation performances under different cooling speed. The F-value and *p*-value were shown in Table S3. For recovery ratio and actuated speed, there were no significant difference between groups. However, the fixed force and actuated force of forced air cooling were significantly stronger when compared to natural cooling.

### 3.4. The Effect of Idling Time on Shape Memory Properties and Actuation Performances

**Figure 5:**
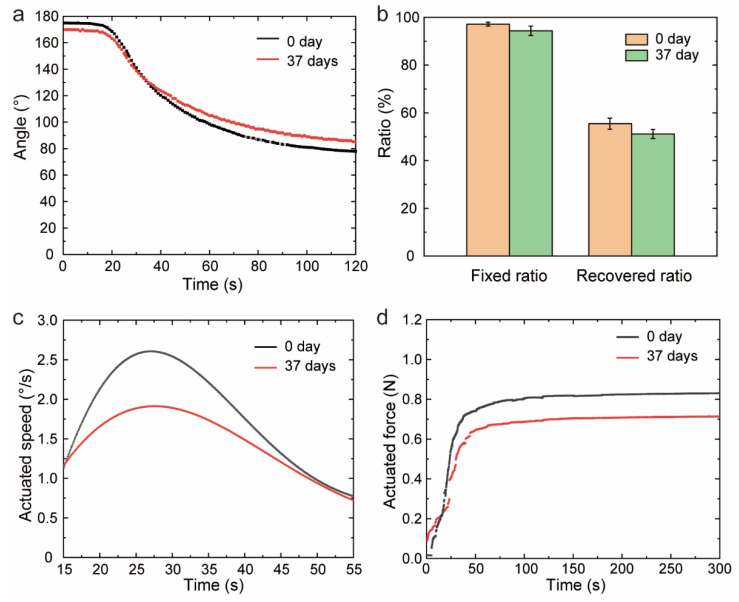
The effect of idling time on shape memory properties and actuation performances. (**a**) The variation in angle for the different idling time. (**b**) The shape memory properties for different idling time. (**c**) The angular speed for the different idling time. (**d**) The recovery force for different idling time.

Considering the advantages of shape memory PEEK, we envisaged PEEK composites as a deployable structure for aerospace devices, which needed to work at the right time after installation. Namely, the temporarily fixed composites would not be used immediately. For example, the solar sail of PW-Sat2 CubeSat designed by Warsaw University of Technology was deployed 26 days after launch [46]. Therefore, another important factor-idling time that had been neglected in previous studies on SMPs was taken into account. Idling time was the waiting time between the shape fixed process and the recovery process. Considering the pre-launch activities, such as transporting and assembling, we tested the shape memory properties and actuation performances with an idling time of more than 1 month (37 days). Firstly, several samples were fixed to the same temporary shape under the same condition for 2 min. Then, some of them were heated above the glass transition temperature after the fixed process immediately, while others were heated up after 37 days. The recovery of angle and speed were characterized in Figure 5a,b. The fixed shape slightly recovered to the original shape at room temperature after 37 days, giving rise to 2.8% decrease in the fixed ratio. Meanwhile, the recovered angle had diminished, and the recovery ratio decreased by 7.7%. Moreover, the actuated capacities shown in Figure 5c,d were distinguishing. The angular speed of the sample with a short idling time was fast during the whole process, and the maximum speed was 1.4 times that of the sample with a long idling time. The recovery force after 37 days decreased by 15% leading to a weaker actuated capacity. Moreover, we conducted the one-way ANOVA for different groups, and the F-value and *p*-value were shown in Table S4. For recovery ratio and actuated speed, there were no significant difference between groups. Namely, deployable construction made of PEEK composites would have a reliable working accuracy for aerospace field. However, the fixed force and actuated force with short idling time were significantly more strongly when compared to that with long idling time. This might limit the application of PEEK actuator.

### 3.5. The Effect of Structure Parameters on Shape Memory Properties and Actuation Performances

The performances had been improved by external conditions in the shape memory process. Moreover, the structure of the sample would affect mechanical behavior in bending deformation. We studied the shape memory behavior of PEEK from two dimensions; arc length and thickness.

**Figure 6:**
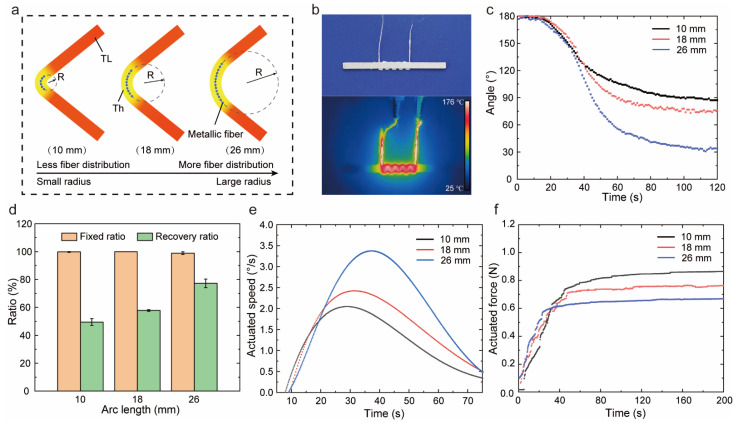
The effect of actuated length on shape memory properties and actuation performances. (**a**) The sketch of different actuated lengths in bending deformation. (**b**) The thermal imaging and optical photograph of sample with actuated length 18 mm. (**c**) The variation in angle for different actuated lengths. (**d**) The shape memory properties for different actuated lengths. (**e**) The angular speed for different actuated lengths. (**f**) The recovery force for different actuated lengths.

In Figure 6a, the wire path with 2 mm spacing was configurated on the centerline. The wire embedded in the interlayer was designed to control the arc length of bending behavior by adjusting the number of paths. When the samples were identically bent 180° by tangential force applied at the top, the sample with more paths had a larger curvature radius. The photo and thermal map of the sample with ten wire paths were demonstrated in Figure 6b, and the heated length was consistent with the length of path distribution. Three kinds of samples were shaped under the same conditions, and the angular recovery was recorded in Figure 6c. Samples with a longer arc length had a greater recovered angle, and the recovery ratio was summarized in Figure 6d. The recovery ratio of samples with an arc length of 26 mm was 77%, while the sample with an arc length of 10 mm has a recovery ratio of only 49%. When the samples were bent at a small curvature radius, the applied strain per unit arc length was larger, leading to a more serious slip for molecular chains. Such an irrecoverable slip meant more plastic deformation in bending behavior. Therefore, the recovered angle was small and possessed a low shape recovery ratio for the sample with a long arc length. Moreover, the samples with a curvature radius of 26 mm recovered fast due to a better elastic deformation capability. In Figure 6e, the maximum speed of the samples with a curvature radius of 26 mm was 3.16 °/s, which was 1.3 times that of the samples with a curvature radius of 10 mm. Moreover, the recovery force was tested in Figure 6f. As the curvature radius increased, the actuated force decreased. This was interpreted as the more the stored strain energy per unit arc length, the greater the force triggered by the elastic recovery process. We conducted the one-way ANOVA for different groups, and the F-value and *p*-value were shown in Table S5. For actuated speed, there were no significant difference between groups. However, the recovery ratio, fixed force and actuated force were significantly influenced by actuated length.

**Figure 7:**
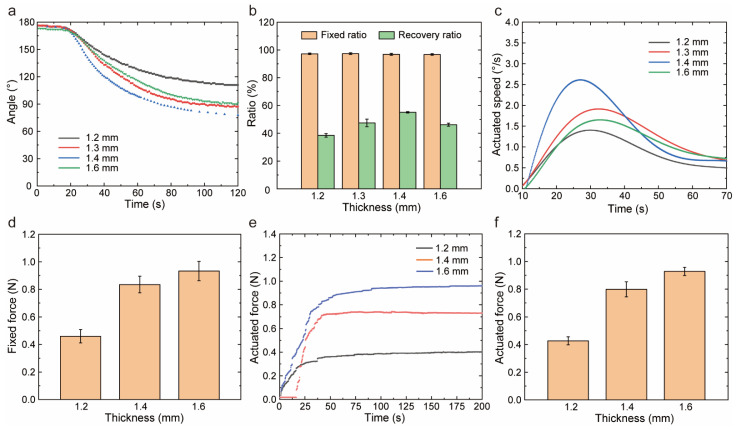
The effect of thickness on shape memory properties and actuation performances. (**a**) The angular recovery for different thicknesses. (**b**) The shape memory properties for different thicknesses. (**c**) The angular speed for different thicknesses. (**d**) The maximum speed for different thicknesses (**e**) The fixed force for different thicknesses. (**f**) The recovery force for different thicknesses.

Another dimension, thickness, influenced the bending mechanical performances of the cross-section. We studied the effect of thickness on shape memory performances in Figure 7a–d. Initially, the recovered angle and recovery speed increased as the thickness of the samples increased. This was interpreted as the thicker samples with more volume could store more strain energy. Thus, the thicker samples released more strain in the recovery stage, showing a faster recovery within a certain period. For every 0.1 mm increase in thickness, the shape recovery ratio increased by 15%, while the maximum speed increased by 35%. When the thickness was 1.6 mm, abnormal behaviors were observed. The recovery angle and recovery speed of the samples with a thickness of 1.6 mm were smaller than that of the samples with a thickness of 1.4 mm. Since the heat transfer was difficult in thick samples, especially for high-performance PEEK with thermal isolation property, the recovery speed was restrained significantly leading to a weak shape recovery ratio. In Figure 7e,f, the recovery force increased with thickness similarly to the fixed force. This was because the thicker samples have a larger moment of inertia of the section, which came into being a larger bending strength. Thus, whether in the fixed or recovered process, the thicker samples showed greater force. The relationship between thickness (*d*) and fixed force (*F_f_*)was analyzed by linear regression analysis with R^2^ = 0.794, and shown as Equation (5). When the thickness of samples increased from 1.2 mm to 1.6 mm, the recovery force increased by 117%. The relationship between thickness and recovery force was analyzed by linear regression analysis with R^2^ = 0.856, and shown as Equation (6). The actuated performances of PEEK were improved effectively by increasing the thickness. We conducted the one-way ANOVA for different groups, and the F-value and *p*-value were shown in Table S6. All shape memory properties and actuation performances had significant difference when the thickness changed.

(5) $$F_{f}=1.18d-0.91$$

(6) $$F_{r}=1.25d-1.04$$

### 3.6. Weight Transport and Deployable Structure of Shape Memory PEEK

**Figure 8:**
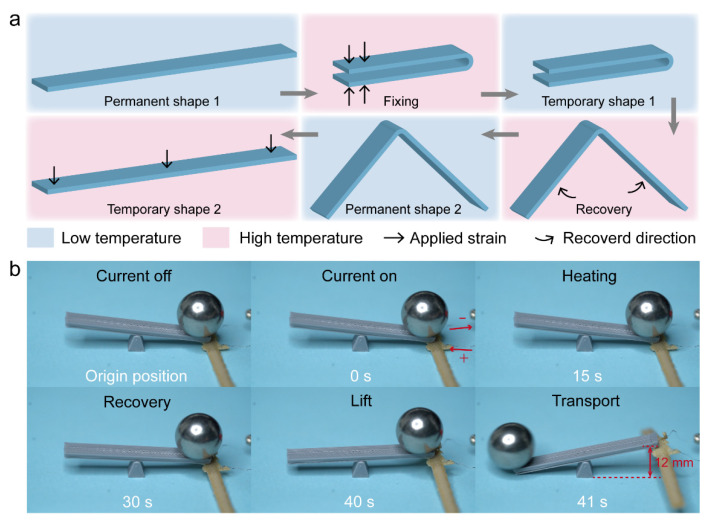
Weight transport by shape memory PEEK. (**a**) The programming of actuated PEEK sample. (**b**) The experiment process of weight transport.

To demonstrate the actuated capacity of PEEK, a sample with a temporary shape was designed as a conveyor to lift and transport a weight. The printed sample embedded with wire was 1.3 mm in thickness with a 10 mm actuated arc length. Firstly, the printed sample (permanent shape 1) was fixed at 180° at a high temperature to freeze the temporary shape 1 in Figure 8a. Due to its shape memory effect in the first cycle, the sample recovered free to about 90° after electric actuation, called permanent shape 2. Then, the sample was fixed again from 90° (permanent shape 2) to 0° (temporary shape 2) at a high temperature. This state was regarded as an actuated shape, which was suitable for lifting and transporting the weight. A seesaw was designed additionally for transporting a 17 g metallic ball in Figure 8b and Video S2. The metallic ball was lifted gradually by the electric PEEK up to 12 mm high within 40 s. Meanwhile, the metallic ball went down the seesaw to the other side and was transported for a distance.

**Figure 9:**
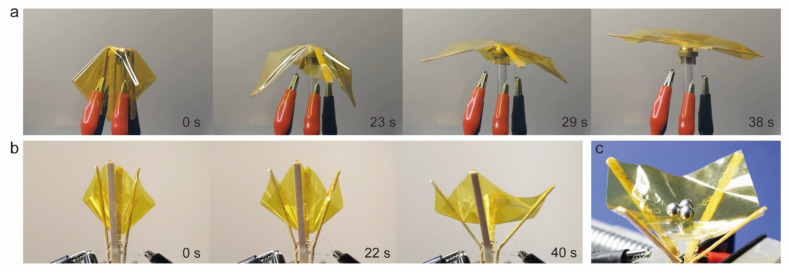
The deployable structure of shape memory PEEK. (**a**) An experimental simulation of deployed drag sail. (**b**) A mimetic catcher with deployed joints. (**c**) The expanding film capturing and loading the stuff.

An additionally designed application prospect for shape memory PEEK was a deployable structure embedded with wire. The deployable structure is widespread in aerospace, where almost all large-size structures are deployable. Therefore, we demonstrated two deployable structures to enrich the promise of shape memory PEEK. An experimental simulation of deployed drag sail was shown in Figure 9a and Video S3. A polyimide film with 15 μm thickness was adhered to the surface of PEEK samples and gradually spread as the shape memory recovery within 40 s. Another deployed structure was a catcher with flexional joints in Figure 9b. The expanding film was cambered for capturing and loading the stuff in Figure 9c. This was expected to be applied to space junk capture. These two deployable structures were all tested five times, and kept the same performance.

## 4. Conclusions

In this work, high-temperature PEEK embedded with wire was manufactured by composite 4D printing for combining the SMP and stimuli in actuation technology. Integrated shape memory PEEK was expected to play a substitute role in harsh environments, such as aerospace. The stable and consistent shape memory properties were verified in multiple shape memory deformation, representing great working capacities and deformation accuracy of PEEK actuators. Based on these, we investigated the shape memory properties and actuation performances from applied conditions and structure parameters of samples. Moreover, a conveyor and a deployable drag sail were mimicked, expanding the potential of transportation and deformation. The lightweight structure and non-motor portability showed a breakthrough in special environments. This study paves the way in front of designing and optimizing the future electro-active PEEK actuators in aerospace.

## Data Availability

Data is contained within the article or supplementary materials.

## Supplementary Materials

The following supporting information can be downloaded at: https://www.mdpi.com/article/10.3390/polym14183800/s1, Figure S1: The shape memory properties and actuation performances of PEEK for fifty cycles; Figure S2: The surface topography of PEEK after shape memory cycles; Figure S3: The electrothermal effect of Cr_20_Ni_80_ wire; Table S1: The properties of Cr_20_Ni_80_ wire; Table S2: The one-way ANOVA for repeated measures of shape memory properties and actuation performances under different heating temperatures; Table S3: The one-way ANOVA for repeated measures of shape memory properties and actuation performances under different cooling speeds; Table S4: The one-way ANOVA for repeated measures of shape memory properties and actuation performances under different idling times; Table S5: The one-way ANOVA for repeated measures of shape memory properties and actuation performances under different actuated lengths; Table S6: The one-way ANOVA for repeated measures of shape memory properties and actuation performances under different thicknesses; Video S1: The measurement of the force; Video S2: Weight Transport by PEEK; Video S3: Deployable structure of PEEK.
