# Supplementary material for: The Shape Memory Properties and Actuation Performances of 4D Printing Poly (Ether-Ether-Ketone)
Source: Polymers (Basel). 2022 Sep 11;14(18):3800. doi: 10.3390/polym14183800 (PMC9502534; doi:10.3390/polym14183800)
Supplement: Supplementary file 1 [file polymers-14-03800-s001.zip › polymers-1893125-supplementary.pdf]

Supporting Information

The Shape Memory Properties and Actuation Performances of 4D Printing Poly(ether-ether-ketone)

Yuting Zhou, Luquan Ren, Jianfeng Zang, Zhihui Zhang**

1. The Key Laboratory of Bionic Engineering (Ministry of Education) and the College of Biological and Agricultural Engineering, Jilin University, 5988 Renmin Street, Changchun 130025, China

2. School of Optical and Electronic Information and Wuhan National Laboratory for Optoelectronics, Huazhong University of Science and Technology, Wuhan 430074, China

3. The State Key Laboratory of Digital Manufacturing Equipment and Technology, Huazhong University of Science and Technology, Wuhan 430074, China

*Corresponding author. Email: jfzang@hust.edu.cn; zhzh@jlu.edu.cn;

Table S1. The properties of $Cr_{20}Ni_{80}$ wire.

Parameters	Diameter (mm)	Resistance per meter (Ω/m)	Tensile strength (N/mm^2)	Density (g/cm^3)
Value	0.05	5.551	750	8.4

Table S2. The one-way ANOVA for repeated measures of shape memory properties and actuation performances under different heating temperatures.

Factors	F-value	p-value	Significance level
Recovery ratio	3.14991	3.14991	-
Actuated speed	1.49829	0.29663	-
Fixed force	119.94	1.45306E-5	**
Actuated force	92.8	3.06747E-5	**

Note: - represents $p>0.05$; * represents $0.01<p\leqslant 0.05$; ** represents $p\leqslant 0.01$

Table S3. The one-way ANOVA for repeated measures of shape memory properties and actuation performances under different cooling speeds.

Factors	F-value	p-value	Significance level
Recovery ratio	3.84803	0.12133	-
Actuated speed	1.49829	0.29663	-
Fixed force	119.94	1.45306E-5	**
Actuated force	92.8	3.06747E-5	**

Table S4. The one-way ANOVA for repeated measures of shape memory properties and actuation performances under different idling times.

Factors	F-value	p-value	Significance level
Recovery ratio	5.04829	0.08795	-
Actuated speed	6.13041	0.06851	-
Fixed force	8.01265	0.04731	*
Actuated force	13.11584	0.00849	**

Table S5. The one-way ANOVA for repeated measures of shape memory properties and actuation performances under different actuated lengths.

Factors	F-value	p-value	Significance level
Recovery ratio	112.31183	1.76093E-5	**
Actuated speed	4.12839	0.07454	-
Fixed force	22.22355	0.00168	**
Actuated force	7.33009	0.02449	*

Table S6. The one-way ANOVA for repeated measures of shape memory properties and actuation performances under different thicknesses.

Factors	F-value	p-value	Significance level
Recovery ratio	15.18799	0.00115	**
Actuated speed	14.31882	1.50891E-4	**
Fixed force	80.7625	6.73539E-7	**
Actuated force	130.19643	1.14258E-5	**

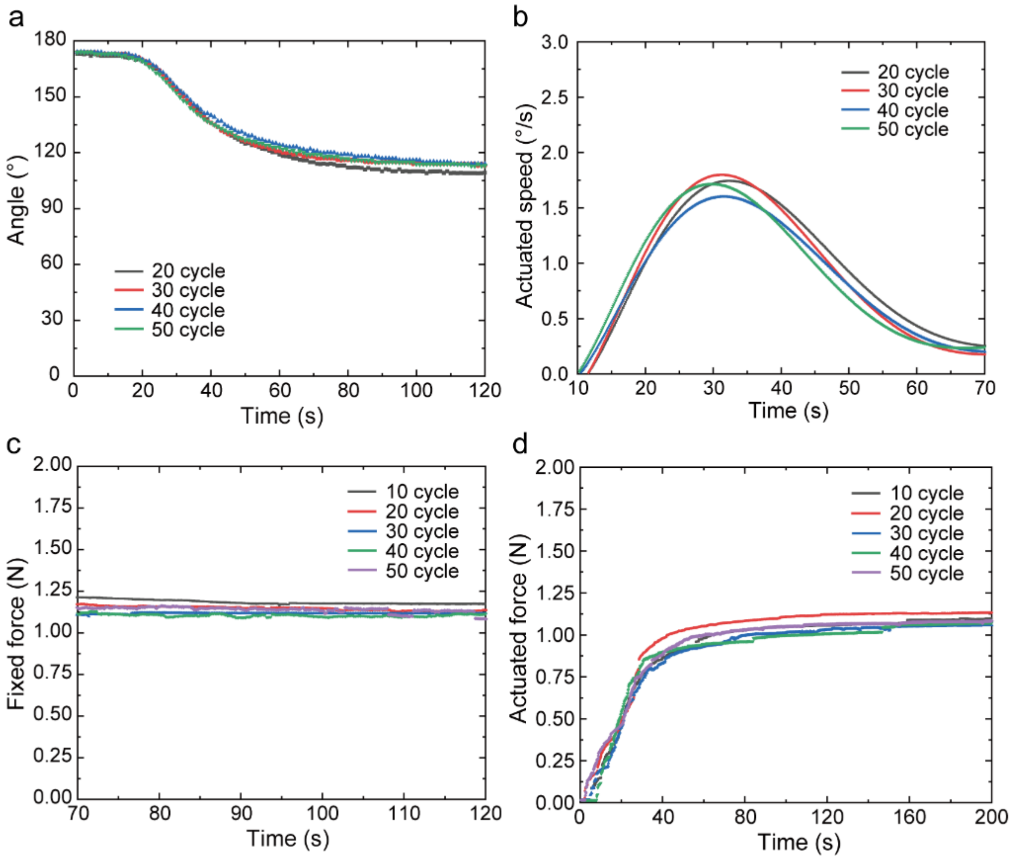

Figure S1. The shape memory properties and actuation performances of PEEK for fifty cycles. a, The variation of angle in shape recovery process. **b**, The angular speed in shape recovery process. **c**, The fixed force in shape fixed process. **d**, The recovery force in shape memory recovery.

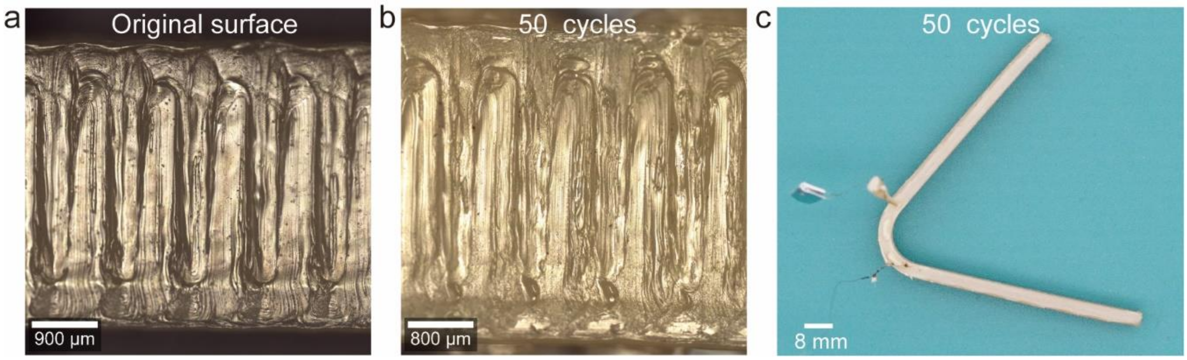

Figure S2. The surface topography of PEEK after shape memory cycles. a, The surface topography of the original surface. b, The surface topography after 50 cycles. c, The surface topography in the direction of layer thickness.

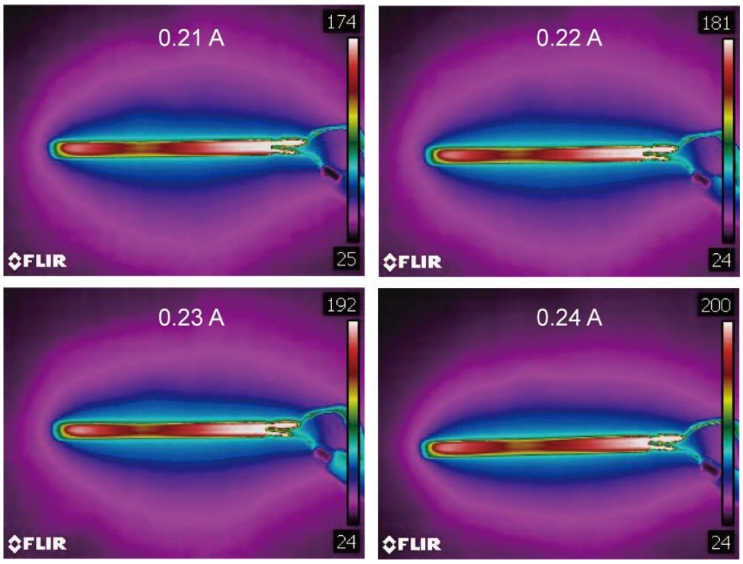

Figure S3. The electrothermal effect of $Cr_{20}Ni_{80}$ wire.

Video S1. The measurement of the force. The composite PEEK sample was triggered by a power wire, while a pressure sensor fixed on the supporting structure was connected with a source measure unit to successively detect the pressure.

Video S2. Weight Transport by PEEK. A 17 g metallic ball was lifted gradually by the electric PEEK up to 12 mm high within 40 s.

Video S3. Deployable structure of PEEK. A simulative deployed drag sail gradually spread as the shape memory recovery.
